# Efficacy of Single-Dose Del Nido Cardioplegia Beyond 90 Minutes in Adult Cardiac Surgery

**DOI:** 10.3390/jcm14072248

**Published:** 2025-03-26

**Authors:** Murat Yücel, Emrah Uğuz, Kemal Eşref Erdoğan, Erol Şener

**Affiliations:** 1 Department of Cardiovascular Surgery, Ankara Bilkent City Hospital, 06800 Ankara, Türkiye; 2 Department of Cardiovascular Surgery, Ankara Yıldırım Beyazıt University Faculty of Medicine, 06010 Ankara, Türkiye; emrahuguz@gmail.com (E.U.); kemal_esref@hotmail.com (K.E.E.); drerolsener@gmail.com (E.Ş.)

**Keywords:** cardioplegia, del Nido cardioplegia, ischemia–reperfusion injury, myocardial protection

## Abstract

**Background:** Del Nido (DN) cardioplegia is widely used in cardiac surgery for its efficacy in providing myocardial protection for up to 90 min with a single dose. However, its safety and efficacy during prolonged ischemia remain unclear. **Methods:** This retrospective study analyzed 471 patients who underwent cardiac surgery with CPB between January 2019 and September 2024. Patients were divided into two groups: ACC durations of 60–90 min (Group A, n = 240) and >90 min (Group B, n = 231). The perioperative characteristics, clinical outcomes, and biochemical markers were compared to evaluate the impact of prolonged ischemia. **Results:** Patients in Group B exhibited significantly higher postoperative troponin I and lactate levels at 4 h post-CPB, suggesting increased myocardial and metabolic stress. Lactate levels normalized within 24 h, indicating transient myocardial dysfunction. Defibrillation requirements and vasoactive inotropic score (VIS) were also significantly elevated in Group B, reflecting compromised myocardial electrical stability and hemodynamic challenges. However, the long-term outcomes such as mortality, LCOS, and MODS showed no significant differences between the groups. **Conclusions:** While DN cardioplegia provides sufficient myocardial protection for ACC durations within 90 min, its efficacy diminishes during prolonged ischemia, as evidenced by increased myocardial injury and hemodynamic instability. Tailored strategies, including standardized redosing protocols and enhanced perioperative management, are essential for optimizing outcomes in complex surgeries with extended ischemia times. Further prospective studies are needed to refine these protocols and assess alternative solutions for myocardial protection.

## 1. Introduction

Myocardial protection is a critical component of cardiac surgeries requiring cardiopulmonary bypass (CPB), directly influencing surgical success and clinical outcomes. Cardioplegia solutions, the cornerstone of myocardial protection strategies, induce cardiac arrest by depolarizing the extracellular membrane potential, thereby creating a bloodless surgical field [1].

Ischemia–reperfusion injury, driven by reactive oxygen species and calcium ions, is the primary determinant of myocardial damage during cardiac surgery. Del Nido (DN) cardioplegia, initially developed by Dr. Pedro del Nido in the 1990s, has gained widespread acceptance in pediatric and adult cardiac surgery due to its unique formulation. The solution, a 1:4 blood–crystalloid mixture with low calcium and high potassium levels, provides prolonged myocardial protection by reducing intracellular calcium influx owing to its potassium-induced depolarization and lidocaine-mediated sodium channel blockade [2,3,4].

DN cardioplegia’s main advantage is its ability to offer effective myocardial protection for 60–90 min with a single dose, which aligns with the duration of standard cardiac surgeries [5,6]. However, in cases in which the aortic cross-clamp (ACC) time exceeds this optimal window, its protective capacity may diminish, potentially leading to adverse biochemical and clinical outcomes [1,7,8]. Despite its increasing use in adult cardiac surgery, there is limited prospective and retrospective evidence evaluating the safety and efficacy of DN cardioplegia beyond the 90 min threshold [9].

This study aims to address this gap by comparing patients undergoing cardiac surgery with ACC times within (60–90 min) and beyond (>90 min) the optimal duration. Specifically, it evaluates the biochemical, mechanical. and clinical differences between these groups and investigates whether additional myocardial protection strategies, such as redosing, are warranted for extended ACC times.

## 2. Materials and Methods

### 2.1. Patient Selection

This study was approved by the Institutional Ethics Committee (Approval No: E2-23-4060). Informed consent was not required due to the retrospective design of the study.

**Figure 1:**
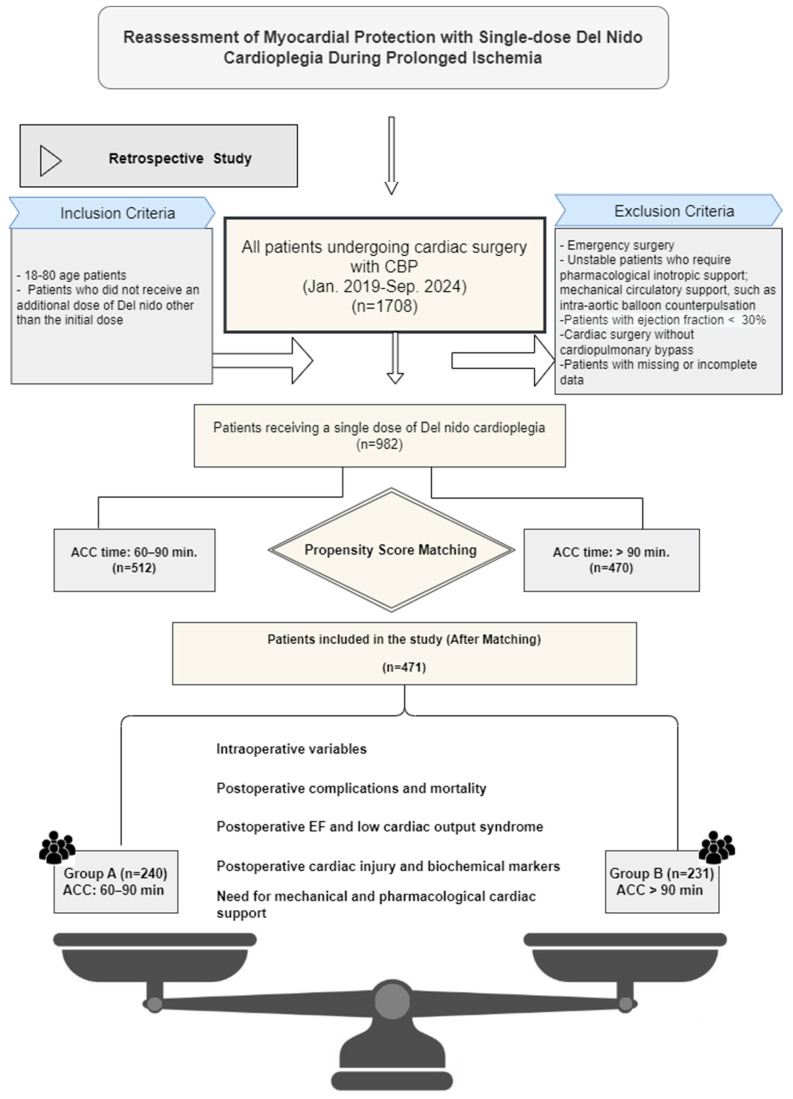
Graphical representation of the study design for patients undergoing cardiac surgery using a single dose of del Nido (DN) cardioplegia with aortic cross-clamp times of 60–90 min and 90 min or more. The number of patients was reduced to 471 using propensity score matching.

This study evaluated a total of 1708 patients who underwent cardiac surgery under cardiopulmonary bypass (CPB) using del Nido cardioplegia between January 2019 and September 2024. The study design and inclusion and exclusion criteria are shown in Figure 1. After applying the inclusion and exclusion criteria, the remaining 982 patients were included in our study and divided into two groups according to the duration of ACS (ACS duration 60–90 min and >90 min). The propensity score matching (PSM) method was applied to minimize the main demographic and clinical differences between the groups. After PSM, a total of 471 patients were matched to Group A, ACS: 60–90 min (n = 240) and Group B, ACS > 90 min (n = 231). The matching process was performed using the many-to-one matching method. Patients in each treatment group were assigned one or more control patients with the closest propensity score. The caliper width was set at 0.2 standard deviations (SDs). A multivariable logistic regression model was used for matching. Age, gender, body mass index (BMI), and comorbidities (hypertension, diabetes mellitus, chronic kidney disease) were included in the model. After matching, a standardized mean difference (SMD) ≤ 0.1 criterion was used to assess the equivalence level of the groups, and it was confirmed that a statistically acceptable balance was achieved between the groups.

### 2.2. Myocardial Protection

This study included patients who received only a single dose of DN cardioplegia. Those who required additional doses for any reason, regardless of the aortic cross-clamp (ACC) duration, were excluded (Figure 1).

The DN cardioplegia solution was prepared by perfusionists according to institutional protocols and the original formulation. It was delivered as a 1:4 blood–crystalloid mixture at 4–8 °C, with a dose of 20 mL/kg, over 5 min at a pressure of 80–100 mmHg. Cardioplegia was administered via antegrade, retrograde, or combined routes, with the choice based on surgical complexity and patient anatomy. The ACC time was continuously monitored, and the surgical team was informed when it exceeded 90 min. In cases with no observed electrical or mechanical activity, the procedure was continued without redosing at the discretion of the surgeon.

### 2.3. Data Collection and Definitions

Preoperative demographic characteristics (age, sex, BMI), cardiovascular risk factors, and comorbidities were collected for comparison between the groups. Intraoperative variables included the type of surgery, CPB duration, and ACC duration. Postoperative outcomes included drainage volume, mechanical ventilation time, ICU stay, hospital stay, and early complications (e.g., infections and acute kidney injury). Only postoperative lactate levels were categorized into the early and late phases. Early lactate levels were measured within the first 4 h, and late lactate levels were obtained at the 24th hour postoperatively. Other biochemical markers and biomarkers of cardiac injury were not available for the first 4 h postoperatively in some patients. Therefore, only values recorded within the first 24 h were included in the analysis. Key endpoints included postoperative inotropic and mechanical cardiac support requirements defined by the use of IABP and ECMO. LCOS was defined as a systolic blood pressure < 90 mmHg and a cardiac index < 2 L/min/m^2^ requiring inotropic support. Inotropic administration was defined as the dose of dopamine, dobutamine, epinephrine (E), norepinephrine (NE), or milrinone during the first 24 h postoperatively, and the amount of cardiovascular support was calculated using the vasoactive–inotropic score (VIS) formula [10]. Standard clinical criteria were applied for other complications.

### 2.4. Statistical Analysis

Descriptive statistics were used to summarize the data as means, standard deviations, medians, frequencies, and percentages. The normality of data distributions was assessed using the Kolmogorov–Smirnov and Shapiro–Wilk tests. Independent sample *t*-tests were used for normally distributed variables, and Mann–Whitney U tests were applied for non-normally distributed variables. Categorical variables were analyzed using the chi-squared or Fisher’s exact test. Effect sizes for statistically significant differences were determined using Cohen’s d and reported along with *p*-values to provide a more comprehensive assessment of clinical impact. Statistical significance was set at *p* < 0.05. Analyses were conducted using SPSS 27.0 software.

## 3. Results

### 3.1. Preoperative Characteristics

The preoperative patient characteristics are summarized in Table 1. A total of 471 patients were divided into two groups based on ACC duration: Group A (60–90 min, n = 240) and Group B (>90 min, n = 231). The mean patient age was 57 years, with no significant differences in age, sex, BMI, smoking history, atrial fibrillation prevalence, or comorbidities between the groups (*p* > 0.05).

### 3.2. Perioperative and Procedural Characteristics

Perioperative data and surgical distributions are presented in Table 2. The total CPB time and mean ACC time were significantly higher in Group B (*p* < 0.0001). The intraesophageal temperature was significantly lower in Group B (*p* < 0.0001). The initial cardioplegia dose was higher in Group B; however, this difference was not statistically significant (*p* > 0.05).

Defibrillation was required less frequently in Group A, whereas temporary pacing rates were similar between the groups. The majority of the surgical procedures were CABG, and there were no significant differences in procedural characteristics or cardioplegia delivery routes between the groups.

### 3.3. Early Postoperative and Clinical Results

**Figure 2:**
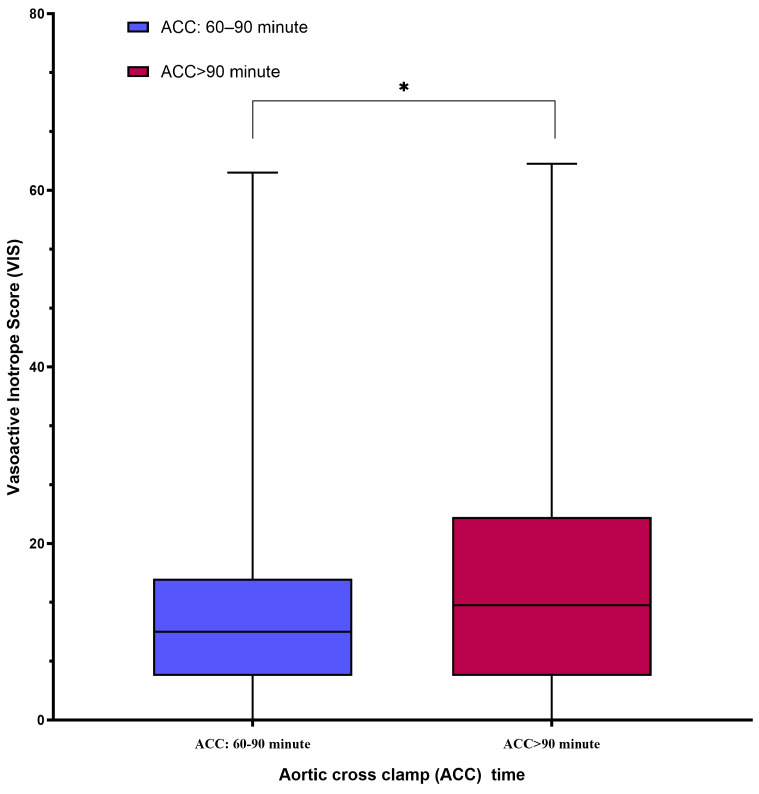
The box-plot graph comparing postoperative vasoactive–inotropic score (VIS) between the groups showed that VIS, reflecting cumulative inotropic support, was significantly higher in Group B. * *p* < 0.05.

The postoperative clinical outcomes and early complications are summarized in Table 3. Group B demonstrated a significantly longer ICU stay, extubation time, and total hospital stay than Group A (*p* < 0.05). The vasoactive–inotropic score (VIS), which reflects cumulative inotropic support, was significantly higher in Group B (*p* < 0.05) (Figure 2).

Although the use of IABP and ECMO was more frequent in Group B, these differences were not statistically significant. The number of patients with renal and hepatic dysfunction was higher in Group B; however, the differences were not statistically significant. Early postoperative complications, including LCOS and mortality, showed no significant differences between the groups, and postoperative EF values were similar.

### 3.4. Biochemical and Myocardial Injury Markers

**Figure 3:**
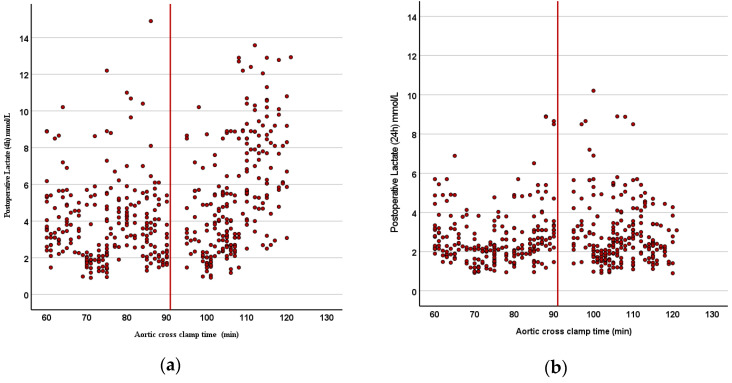
(**a**,**b**) Scatter plot graphs revealing (**a**) early (4 h) and (**b**) late (24 h) postoperative lactate levels: early postoperative lactate levels were significantly higher in Group B (*p* < 0.001), although they equalized within 24 h in both groups.

**Figure 4:**
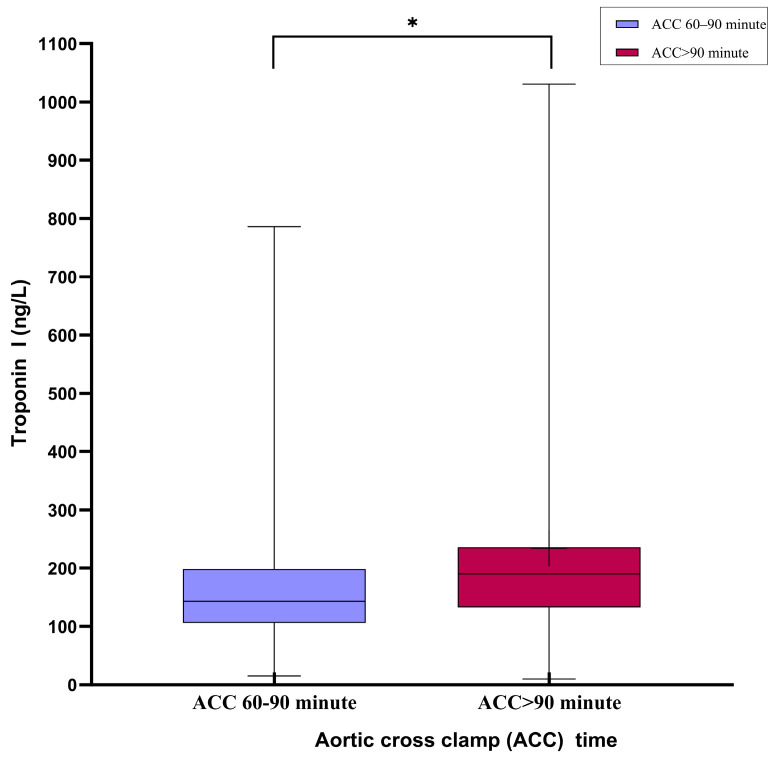
Box-plot graph comparing troponin I (Trop-I) levels in the first 24 h postoperatively in both ischemic time intervals: TnI levels were significantly higher in the group with an aortic cross-clamping time >90 min in the postoperative first 24 h. * *p* < 0.05.

The biochemical parameters and cardiac biomarkers are compared in Table 4. Early postoperative lactate levels were significantly higher in Group B (*p* < 0.001), although they equalized within 24 h in both groups (Figure 3a,b). Postoperative troponin I levels were significantly higher in Group B (*p* < 0.05; Figure 4). The CK-MB levels were also higher in Group B, but the difference was not statistically significant. Other biochemical markers, including pH, urea, creatinine, LDH, AST, ALT, hematocrit, and platelet levels, showed no significant differences between the groups.

To further evaluate the clinical relevance of these differences, effect sizes were calculated using Cohen’s d for the parameters that showed significant differences. The effect size for lactate was 0.55 (indicating a medium effect), while the effect size for troponin I was 0.27 (indicating a small effect).

## 4. Discussion

Del Nido (DN) cardioplegia is a widely accepted myocardial protection strategy in cardiac surgeries due to its ability to provide prolonged ischemic times with a single dose. This simplifies surgical workflow by reducing the need for repeated dosing, minimizing interruptions, and enabling efficient procedures [1,2,3]. However, the safety and effectiveness of DN cardioplegia for aortic cross-clamp (ACC) durations longer than 90 min are still debated, especially its ability to protect the heart during extended periods of ischemia [1,2,3,4].

Existing studies consistently indicate that DN cardioplegia is effective for 60–90-min durations, offering myocardial protection comparable to conventional cardioplegia solutions with smaller volumes and shorter ischemic times [5,6,7,8]. However, there is limited clinical evidence beyond this range. Most research has focused on cases with standard ACC durations or on patients receiving additional doses when ischemia extends beyond 90 min. For example, Sanetra et al. redosed DN cardioplegia at 90 min during aortic valve replacement surgeries [9], while Timek et al. routinely administered a second dose at 60 min during isolated coronary artery bypass grafting (CABG) procedures [8]. These studies highlight the uncertainty surrounding the upper ischemic time limit for a single dose of DN cardioplegia in the adult population.

Animal studies have also provided additional insights. Govindapillai et al. demonstrated that a single dose of DN cardioplegia offers better functional recovery than repeated dosing at 20 min intervals after 60 min of ischemia [10]. Nakao et al. found that ischemic durations of 90 and 120 min had minimal differences in left ventricular recovery in porcine models [11]. These findings suggest that DN cardioplegia may remain effective for up to 120 min in controlled settings [12]. However, clinical translation is complicated by the variability in patient profiles, including myocardial hypertrophy, coronary artery disease, and surgical complexity. This study aimed to evaluate the clinical and biochemical outcomes of DN cardioplegia in patients with ACC durations of 60–90 min compared to those exceeding 90 min. A significant finding was the increased need for defibrillation in the >90 min group, which underscores compromised myocardial electrical stability during prolonged ischemia. This result aligns with prior studies associating extended ischemic intervals with heightened oxidative stress, calcium dysregulation, and ischemia–reperfusion injury, all of which disrupt electrical homeostasis and precipitate ventricular arrhythmias [13,14].

In the >90 min group, significantly elevated vasoactive inotropic scores (VISs) were observed, reflecting the increased reliance on inotropic support to maintain hemodynamic stability during the postoperative period. The VIS serves as a cumulative index of inotropic and vasopressor requirements, making it a critical prognostic indicator in cardiac surgery [15]. High VIS values have been consistently associated with greater morbidity, prolonged ICU stay, and extended mechanical ventilation duration, all of which were evident in our study cohort u (SIRS) [16]. The elevated VIS in the >90 min group is indicative of the heightened metabolic and hemodynamic strain imposed by prolonged ischemic durations, likely stemming from increased oxidative stress, myocardial stunning, and reperfusion injury.

Prolonged ischemic time has been shown to compromise myocardial contractility due to impaired ATP generation, calcium overload, and cellular edema. These pathological changes necessitate aggressive pharmacological support to maintain the systemic perfusion and cardiac output. The correlation between high VIS and postoperative complications, including low cardiac output syndrome (LCOS) and systemic inflammatory response syndrome (SIRS), has been well-documented in the literature [16]. Our findings align with these observations, highlighting the necessity for vigilant hemodynamic monitoring and tailored perioperative strategies in patients anticipated to undergo an extended ACC duration. Furthermore, while VIS levels eventually stabilized postoperatively, the transient period of hemodynamic instability could have contributed to the increased duration of ICU and hospital stays observed in this group. These outcomes underline the importance of optimizing myocardial protection strategies, including reconsideration of redosing protocols, in surgeries with prolonged ischemic times to mitigate metabolic burden and improve recovery trajectories.

Contrary to Guim et al.’s findings [17], which reported comparable defibrillation and postoperative arrhythmia rates even with ACC durations exceeding 120 min, our results reveal that DN cardioplegia’s protective efficacy diminishes when used beyond its optimal duration. Meta-analyses have highlighted DN cardioplegia’s advantage in reducing defibrillation needs compared to conventional solutions [18]. However, our study indicates that this benefit is significantly compromised during prolonged ischemia, suggesting a critical threshold beyond which DN cardioplegia may no longer provide adequate myocardial protection.

Interestingly, despite the increased defibrillation requirements in the >90 min group, the rates of permanent pacemaker implantation and postoperative atrial fibrillation (AF) were similar between the groups. This finding suggests that although prolonged ischemia induces acute disturbances in myocardial electrical stability, it does not necessarily lead to lasting damage to the cardiac conduction system. Nevertheless, it also highlights the complexity of the factors influencing AF and pacemaker dependency, which may extend beyond ischemia–reperfusion dynamics to include systemic inflammation, atrial remodeling, and patient comorbidities.

These findings underscore the need for enhanced strategies when DN cardioplegia is used in extended ACC durations. Standardized redosing protocols tailored to surgical complexity and patient-specific factors may help preserve the advantages of DN cardioplegia while mitigating the risks associated with prolonged ischemia. Further studies are essential to determine the upper limits of DN cardioplegia efficacy and refine myocardial protection strategies for complex and extended surgical procedures.

Elevated troponin I and lactate levels in the >90 min group emphasize the heightened metabolic and structural strain imposed by prolonged ischemia. Troponin I, a sensitive marker of myocardial injury, remained significantly elevated, suggesting persistent cellular damage [19]. In contrast, CK-MB levels did not show significant differences, likely because of its shorter half-life and lower specificity, making it less reliable for assessing prolonged myocardial injury [20,21].

Lactate, a marker of anaerobic metabolism, was notably elevated four hours post-cardiopulmonary bypass (CPB) in the >90 min group, reflecting the metabolic consequences of prolonged ischemia and reperfusion. The transient nature of this elevation, with levels normalizing within 24 h, suggests that the observed myocardial dysfunction was reversible rather than indicative of irreversible damage. This observation aligns with studies demonstrating that ischemia–reperfusion injury initially disrupts mitochondrial function and energy metabolism, leading to lactate accumulation [7,22,23]. As oxygen delivery and mitochondrial function are restored during reperfusion, lactate is metabolized by the liver and kidneys, explaining its rapid normalization in most cases [7,23].

These findings highlight the need for optimized myocardial protection strategies during extended ischemic periods to mitigate metabolic stress and prevent cumulative myocardial injury.

Despite these short-term challenges, no significant differences were observed in key long-term outcomes, including mortality, low cardiac output syndrome (LCOS), and multiple organ dysfunction syndrome (MODS), between the groups. This suggests that DN cardioplegia provides sufficient myocardial protection, even for extended ischemic durations, albeit with short-term hemodynamic and metabolic trade-offs.

Redosing strategies often depend on the discretion of the surgeon and the institutional protocols. In cases involving mitral valve surgery or complex procedures, administering additional doses of DN cardioplegia may disrupt the surgical exposure or workflow. This underscores the need for standardized redosing protocols to balance myocardial protection and surgical efficiency.

In cardiac surgery, while the use of inotropes is often necessary intraoperatively, the surgical procedure performed outside of the aortic cross-clamp (ACC) period can be influenced by various factors such as patient characteristics [24,25]. The group with prolonged ACC times exhibited higher vasoactive–inotropic scores (VISs), which have been associated with increased morbidity, longer ICU stays, and extended durations of mechanical ventilation. Moreover, studies have demonstrated that longer-than-average ACC times are linked to an increased use of inotropic drugs and a greater need for intra-aortic balloon pump (IABP) support [26,27].

These findings emphasize the importance of optimizing hemodynamic stability in the postoperative period to mitigate these risks.

This study highlights the efficacy of DN cardioplegia within the 60–90-min range, while also addressing the potential risks of extending its use beyond this limit. Elevated lactate and troponin I levels, along with increased defibrillation and VIS requirements, highlight the need for caution in prolonged ischemia. These findings suggest the following.

- Standardized redosing protocols, particularly in surgeries anticipated to exceed 90 min of ACC duration.
- Tailored strategies that consider patient-specific factors such as myocardial hypertrophy or coronary artery disease.
- Prospective studies are needed to evaluate the safety and efficacy of dosing intervals and assess alternative cardioplegia solutions for complex cases.

DN cardioplegia remains a reliable myocardial protection strategy for the standard ACC duration, offering significant practical advantages. However, its application in prolonged ischemic times requires careful consideration of the patient and procedural factors. While long-term outcomes appear unaffected, the transient biochemical and hemodynamic challenges observed highlight the importance of individualized approaches and further research to optimize surgical outcomes.

## 5. Study Limitations

This study has several limitations that need to be considered. First, while propensity score matching helped balance the baseline characteristics, the study’s retrospective design still carries the risk of confounding factors.

Although a multivariate logistic regression model was initially used to calculate the propensity scores, a post-matching regression analysis was not performed because the SMD values used in the logistic regression were <0.1. Furthermore, the retrospective design of the study introduced inherent biases, including potential selection bias and unmeasured confounders, despite the application of propensity score matching (PSM). Furthermore, the relatively small sample size may have limited the statistical power to detect smaller but clinically meaningful differences, particularly with regard to postoperative complications such as low cardiac output syndrome (LCOS) and multiple organ dysfunction syndrome (MODS).

Another important limitation is the lack of a control group receiving additional doses of cardioplegia, which makes it difficult to accurately define the upper limit of myocardial protection achieved with DN cardioplegia during cardiac arrest. The increased inotropic requirements and postoperative metabolic burden observed in the >90 min group suggest that prolonged ischemic times may require alternative cardioplegia dosing strategies that should be evaluated in future studies.

Despite the high adherence to institutional protocols, the involvement of multiple surgeons may have introduced variability in intraoperative management and postoperative outcomes, making it difficult to isolate the influence of surgeon-dependent decisions. In particular, differences in inotropic management, cardioplegia administration techniques, and perioperative fluid strategies may have influenced clinical outcomes.

The assessment of myocardial injury markers (CK-MB and troponin I) also presents a limitation. Blood samples were not collected at uniform time intervals, which may have hampered the ability to assess dynamic trends in these biomarkers. Furthermore, the study did not include additional biomarkers, such as BNP or NT-proBNP, which could provide a more comprehensive assessment of myocardial stress. Future studies should include a broader panel of biomarkers to improve our understanding of the mechanisms of myocardial protection.

Finally, this study examined short-term outcomes. Without long-term echocardiographic data, we could not determine whether the heart dysfunction observed in the prolonged ischemia group was temporary or permanent.

## 6. Conclusions

This study shows that del Nido (DN) cardioplegia provides satisfactory myocardial protection for cross-clamp durations of less than 90 min. However, for ischemic durations exceeding 90 min, a single dose of DN cardioplegia does not provide superior myocardial protection and is associated with adverse clinical outcomes.

DN cardioplegia did not significantly affect mortality or major complications such as LCOS and MODS in patients with prolonged cross-clamp times. However, the observed postoperative metabolic and hemodynamic difficulties suggest that a single dose of DN cardioplegia may not be sufficient for prolonged ischemic periods.

A tailored approach should be considered to optimize myocardial protection during prolonged ischemia. Clinicians should evaluate the need for additional cardioplegia dosing strategies in cases of complex surgeries with prolonged periods of ischemia. Future studies should focus on defining the optimal redosing intervals and volumes while investigating additional cardioprotective techniques to enhance myocardial protection during long surgical procedures. These adjustments can play a crucial role in optimizing myocardial protection and improving patient outcomes in long surgical scenarios.

## Figures and Tables

**Table 1:** Preoperative characteristics.

		Unmatched						Matched					
		Group A (n = 512)		Group B (n = 470)				Group A (n = 240)		Group B (n = 231)			
		Mean ± SD	Median	Mean ± SD	Median	*p*	SMD *	Mean ± SD	Median	Mean ± SD	Median	*p*	SMD *
Age		60.08 ± 10.59	56.82	55.05 ± 10.18	57.00	0.912	0.206	57.50 ± 10.57	58.00	57.05 ± 10.94	57.00	0.912	−0.063
Gender	Male	291.00 ± 56.84%		215.00 ± 45.74%		0.808	0.325	147.00 ± 61.25%		144.00 ± 62.34%		0.808	0.025
	Female	221.00 ± 43.16%		255.00 ± 54.26%			0.255	93.00 ± 38.75%		87.00 ± 37.66%			0.055
Weight (kg)		78.05 ± 12.36	77.00	74.05 ± 13.02	78.00	0.437	0.076	77.90 ± 12.97	77.00	79.05 ± 13.01	78.00	0.347	0.056
BMI		33.89 ± 4.38	27.12	27.02 ± 4.25	27.58	0.356	0.241	27.58 ± 4.27	26.82	28.01 ± 4.25	27.58	0.24	−0.063
EF (preop.)		50.08 ± 9.49	51.21	51.00 ± 7.35	50.00	0.286	−0.05	50.89 ± 9.26	50.00	49.78 ± 7.34	50.00	0.274	0.053
HT	n/%	71.00 ± 13.87%		117.00 ± 24.89%		0.182	−0.44	47.00 ± 19.58%		60.00 ± 25.97%		0.098	−0.07
DM	n/%	92.00 ± 17.97%		65.00 ± 13.83%		0.543	0.48	34.00 ± 14.17%		33.00 ± 14.29%		0.476	0.05
HL	n/%	88.00 ± 17.19%		83.00 ± 17.66%		0.156	0.04	58.00 ± 24.17%		48.00 ± 20.78%		0.421	0.42
PAH	n/%	52.00 ± 10.16%		49.00 ± 10.43%		0.483	0.05	32.00 ± 13.33%		27.00 ± 11.69%		0.453	0.45
REDO	n/%	12.00 ± 2.34%		11.00 ± 2.34%		0.241	0.01	4.00 ± 1.67%		9.00 ± 3.90%		0.140	−0.14
COPD	n/%	41.00 ± 8.01%		36.00 ± 7.66%		0.256	0.08	19.00 ± 7.92%		13.00 ± 5.63%		0.422	0.06
Smoke	n/%	79.00 ± 15.43%		74.00 ± 15.74%		0.396	0.04	43.00 ± 17.92%		32.00 ± 13.85%		0.388	0.04
Renal Failure	n/%	19.00 ± 3.71%		6.00 ± 1.28%		0.098	0.42	6.00 ± 2.50%		4.00 ± 1.73%		0.099	0.08
Pre AF	n/%	62.00 ± 12.11%		58.00 ± 12.34%		0.080	0.04	45.00 ± 18.75%		33.00 ± 14.29%		0.476	0.09

ACC, aortic cross-clamp; SD, standard deviation; BMI, body mass index; HT, hypertension; EF, ejection fraction; DM, diabetes mellitus; HL, hyperlipidemia; PAD, peripheral arterial disease; COPD, chronic obstructive pulmonary disease; AF, atrial fibrillation. * Standardized Mean Difference (SMD): after matching, a standardized mean difference (SMD) ≤ 0.1 criterion was used to assess the equivalence of the groups.

**Table 2:** Perioperative data and surgical distributions.

Variable		ACC = 60–90 min (n = 240)			ACC > 90 min (n = 231)			*p*
		Mean ± SD		Median	Mean ± SD		Median	
Intraoperative data								
	Total CPB time (min)	130.17 ± 20.92		130.00	149.98 ± 20.98		150.00	0.000
	ACC time (min)	75.54 ± 9.28		75.00	107.01 ± 6.68		106.00	0.000
	∆(CPB time-ACC time)	54.63 ± 21.64		55.00	42.97 ± 20.16		41.00	0.000
	Temperature (°C)	30.74 ± 4.57		30.50	30.04 ± 1.42		30.00	0.001
	Amount of AC (mL)	1270.00 ± 313.16		1200.00	1266.67 ± 312.34		1200.00	0.748
	Cardioplegia initial dose (mL)	1493.96 ± 285.92		1500.00	1477.71 ± 292.83		1500.00	0.410
	Amount of total cardioplegia (mL)	1493.96 ± 285.92		1500.00	1477.71 ± 292.83		1500.00	0.410
		n	%		n	%		
	Defibrillation requirement	8.00	3.33%		17.00	7.36%		0.040
	Need for pace	3.00	1.25%		7.00	3.03%		0.180
Operative characteristics								
	CABG	86.00	35.83%		73.00	31.60%		
	AVR	22.00	9.17%		14.00	6.06%		
	MVR	19.00	7.92%		20.00	8.66%		
	TVR	4.00	1.67%		3.00	1.30%		
	AVR+MVR+TrR	9.00	3.75%		8.00	3.46%		
	AVR+MVR	17.00	7.08%		19.00	8.23%		0.763
	MVR+TrR	18.00	7.50%		23.00	9.96%		
	Valve surgery+CABG	26.00	10.83%		31.00	13.42%		
	Aort surgery	33.00	13.75%		33.00	14.29%		
	Diğer	6.00	2.50%		7.00	3.03%		
Cardioplegia delivery method								
	AC	131.00	54.58%		134.00	58.01%		0.454
	ARC	109.00	45.42%		97.00	41.99%		

CPB, cardiopulmonary bypass; ACC, aortic cross-clamp; AC, antegrade cardioplegia; ARC, antegrade and retrograde cardioplegia; CABG, coronary artery bypass graft; AVR, aortic valve replacement; MVR, mitral valve replacement; TrR, tricuspid ring.

**Table 3:** Early postoperative and clinical outcomes of cardiac surgery.

		60–90 min (n = 240)		>90 min (n = 231)		*p*
		Mean ± SD	Median	Mean ± SD	Median	
Postoperative variables						
	Mean ICU stay (h)	36.15 ± 19.977	24.00	43.26 ± 22.438	48.00	0.000
	Intubation time (h)	10.51 ± 10.539	8.00	12.26 ± 8.651	8.00	0.000
	Mean hospital stay	8.50 ± 2.763	8.00	9.42 ± 3.199	9.00	0.001
	Drainage(mL)	680.00 ± 258.366	650.00	667.75 ± 236.710	600.00	0.694
VIS (Vasoactive–inotropic support)		11.496 ± 9.3459	10.000	16.081 ± 12.8049	13.000	0.014
EF (postoperative)		46.58 ± 7.984	45.00	44.96 ± 6.609	45.00	0.074
Mechanical cardiac support						
	IABP requirement	10	4.17%	14	6.06%	0.350
	ECMO requirement	3	1.25%	5	2.16%	0.443
Postoperative complications		n	%	n	%	
	RI	14	5.83%	20	8.66%	0.236
	Dialysis	4	1.67%	5	2.16%	0.693
	Acute liver injury	7	2.92%	12	5.19%	0.209
	Stroke	3	1.25%	7	3.03%	0.180
	MODS	6	2.50%	5	2.16%	0.815
	Pneumonia	9	3.75%	13	5.63%	0.334
	Wound infection	7	2.92%	6	2.60%	0.833
	Re-exploration for bleeding	7	2.92%	3	1.30%	0.223
	Re-intubation (respiratory origin)	8	3.33%	5	2.16%	0.439
	Re-hospitalization	2	0.83%	3	1.30%	0.622
	AF	31	12.92%	40	17.32%	0.182
	LCOS	5	2.08%	8	3.46%	0.361
	Mortality	6	2.50%	10	4.33%	0.273

DN, del Nido; HTK, histidine-tryptophan-ketoglutarate; ICU, intensive care unit; ES, erythrocyte suspension; FFP, fresh frozen plasma; TS, thrombocyte suspension; RI, renal injury; MODS, multiple organ dysfunction syndrome; AF, atrial fibrillation; LCOS, low cardiac output syndrome.

**Table 4:** Postoperative biochemical analysis and myocardial injury markers.

		60–90 min (n = 240)		>90 min (n = 231)		*p*
		Mean ± SD	Median	Mean ± SD	Median	
PH						
	pre-CPB	7.40 ± 0.07	7.40	7.40 ± 0.08	7.41	0.518
	post-CPB	7.40 ± 0.06	7.40	7.39 ± 0.06	7.39	0.072
Lactate (mmol/L)						
	pre-CBP	1.96 ± 1.03	1.80	1.90 ± 0.80	1.80	0.933
	post-CBP (4 h)	3.86 ± 2.17	3.46	5.32 ± 3.00	4.78	0.000 *
	post-CBP (24 h)	2.65 ± 1.38	2.24	2.91 ± 1.61	2.47	0.109
Potassium (mmol/L)						
	pre-CBP	4.56 ± 0.78	4.52	4.43 ± 0.74	4.30	0.082
	post-CBP	4.60 ± 0.68	4.60	4.66 ± 0.77	4.60	0.383
Urea (mg/dL)						
	Preoperative	35.83 ± 9.99	36.50	34.91 ± 8.46	35.10	0.374
	Postoperative	43.37 ± 10.00	41.90	42.52 ± 10.46	40.60	0.166
Creatinine (mg/dL)						
	Preoperative	1.00 ± 0.20	1.01	1.05 ± 0.22	1.06	0.063
	Postoperative	1.31 ± 0.57	1.16	1.41 ± 0.68	1.22	0.076
Hematocrit (%)						
	Preoperative	36.60 ± 3.37	36.47	36.79 ± 3.36	37.32	0.443
	Postoperative	26.28 ± 2.18	26.53	26.39 ± 2.03	26.68	0.615
Platelet (/µL)						
	Preoperative	200.23 ± 76.13	193.50	200.64 ± 69.24	203.00	0.807
	Postoperative	149.38 ± 41.31	143.00	148.10 ± 36.93	143.00	0.985
LDH (U/Lt)						
	Preoperative	272.71 ± 85.95	253.00	282.29 ± 83.21	270.00	0.136
	Postoperative	396.45 ± 134.66	368.50	401.94 ± 146.61	374.00	0.762
AST (U/Lt)						
	Preoperative	28.26 ± 10.61	27.00	27.53 ± 11.15	26.00	0.370
	Postoperative	47.18 ± 111.60	29.00	51.19 ± 105.21	31.00	0.302
ALT (U/Lt)						
	Preoperative	32.70 ± 10.23	34.00	34.32 ± 17.68	33.00	0.668
	Postoperative	72.85 ± 199.08	38.00	59.08 ± 117.82	40.00	0.200
Troponin (ng/L)						
	Preoperative	18.90 ± 43.90	11.75	28.56 ± 117.85	12.25	0.597
	Postoperative (24 h)	204.68 ± 212.25	146.00	271.29 ± 275.02	188.00	0.000 *
CK-MB (ng/L)						
	Preoperative	2.68 ± 1.16	2.66	2.98 ± 2.06	2.78	0.560
	Postoperative (24 h)	10.71 ± 4.82	10.63	11.20 ± 5.66	11.09	0.636

CRP, C-reactive protein; LDH, lactate dehydrogenase; AST, aspartate aminotransferase; ALT, alanine aminotransferase; CK-MB, creatine kinase MB isotype. * Effect sizes were calculated using Cohen’s d.

## Data Availability

The raw data supporting the conclusions of this article will be made available by the authors on request.
